# Foreign Bodies in Pediatric Otorhinolaryngology: A Review

**DOI:** 10.3390/pediatric16020042

**Published:** 2024-06-19

**Authors:** Ivan Paladin, Ivan Mizdrak, Mirko Gabelica, Nikolina Golec Parčina, Ivan Mimica, Franko Batinović

**Affiliations:** 1 Department of ENT and Head and Neck Surgery, University Hospital of Split, Spinciceva 1, 21000 Split, Croatia; imizdrak@kbsplit.hr (I.M.); mgabelica@kbsplit.hr (M.G.); nikolina.golec007@gmail.com (N.G.P.); fbatinovic1@gmail.com (F.B.); 2 Department of ENT, General Hospital Sibenik, 22000 Sibenik, Croatia; mimica.ivan@gmail.com

**Keywords:** foreign bodies, pediatric otorhinolaryngology, bronchoscopy, esophagoscopy

## Abstract

Foreign bodies (FBs) in pediatric otorhinolaryngology represent up to 10% of cases in emergency departments (ED) and are primarily present in children under five years old. They are probably the result of children’s curiosity and tendency to explore the environment. Aural and nasal FBs are the most common and accessible, and the removal methods differ depending on the exact location and type of FB, which can be organic or inorganic. A fish bone stuck in one of the palatine tonsils is the most common pharyngeal FB. Laryngopharyngeal FBs can obstruct the upper respiratory tract and thus become acutely life-threatening, requiring an urgent response. Aspiration of FBs is common in children between 1 and 4 years old. A history of coughing and choking is an indication of diagnostic and therapeutic methods to rule out or confirm a tracheobronchial FB. Regardless of the availability of radiological diagnostics, rigid bronchoscopy is the diagnostic and therapeutic method of choice in symptomatic cases. Radiological diagnostics are more significant in treating esophageal FBs since most are radiopaque. Flexible or rigid esophagoscopy is a successful method of removal. A delayed diagnosis, as with tracheobronchial FBs, can lead to fatal consequences.

## 1. Introduction

Foreign bodies (FBs) account for an average incidence of 7.9% to 9.5% of cases in the otorhinolaryngological ED [1,2,3] and are predominantly common in the pediatric population below five years of age [4,5,6,7]. Their prevalence is high and varies between 57% and 80% [5,8], probably due to the curious nature of children and their tendency to explore the environment [4,8]. Children with developmental psychological disorders, especially from the spectrum of hyperkinetic disorders, as well as those with congenital malformations, are more prone to FBs [7,9,10]. The nose and ear are the most common sites of FBs [11,12]. In contrast, FBs of the lower respiratory tract and esophagus occur less often. Still, due to their seriousness, they require much greater involvement from health professionals in diagnosis and treatment. There are numerous review articles on FBs in children, but none cover all anatomical sites that fall under the care of otorhinolaryngologists. Therefore, the purpose of this article is to present a review of the literature on FBs in pediatric otorhinolaryngology.

## 2. Aural Foreign Bodies

**Figure 1:**
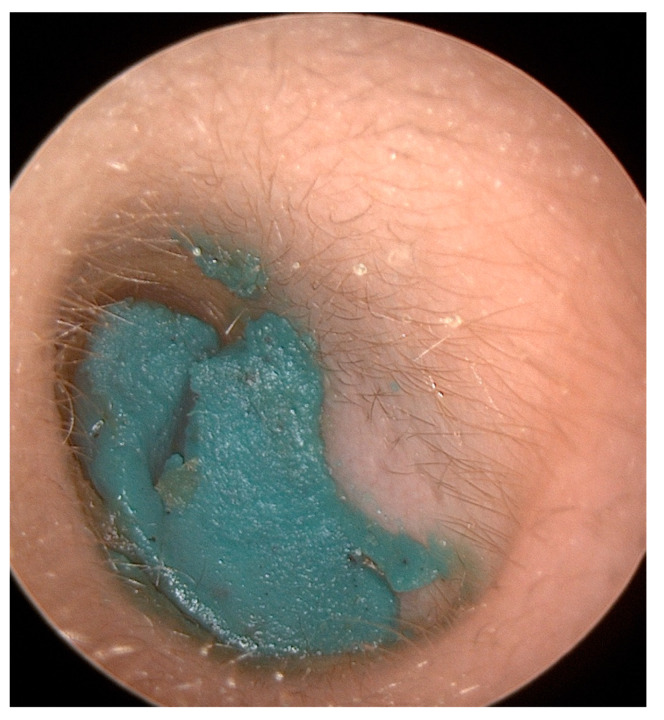
Plasticine found by otoscopy in the left external auditory canal of an 11-year-old boy was successfully removed by aspiration and lavage.

**Figure 2:**
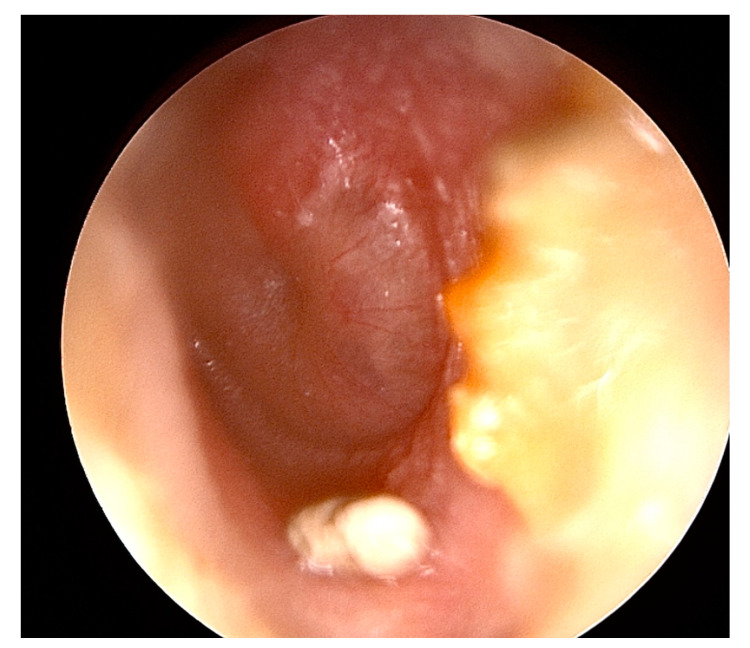
Incidental otoscopic finding of a stone in the left external auditory canal of a 5-year-old boy that was successfully removed by aspiration along with visible cerumen.

FBs in the external auditory canal (EAC) are frequent presentations at an otorhinolaryngological ED (Figure 1 and Figure 2). The median age is six years [9,13,14], with most children younger than eight years [11,13]. There is a male predominance [11,15], and the most common FBs are beads, pebbles, toy parts and paper [11,13]. The most usual presentation is local pain followed by a verbal admission from the child, an incident witnessed by the caregiver, bleeding from the ear, ear discharge, tinnitus, hearing loss and fever [11,16].

Most FBs can be removed without anesthesia or under local anesthesia [16,17]. Yet some FBs require removal under general anesthesia. Tools for the successful removal of objects from the EAC include auricular specula, aspirators, magnets, hooks, forceps and irrigation [18]. Balloon-tipped catheters and adhesive-tipped probes should be used with caution as a method of removing aural FBs in younger children due to higher failure rates and complications [19]. Irrigation should be avoided if there is suspicion of perforation in the tympanic membrane. Particular caution is also required when removing organic FBs due to their swelling in the presence of water, which makes them very difficult to remove [18].

Insects are also quite common, mainly in patients older than ten, and have usually crawled into the ear canal [13,20]. The presentation might be the sensation of intense pain and movement in the ear canal. Management requires killing the insect by instilling alcohol, mineral oil or lidocaine into the ear canal before attempting removal. This should be avoided if there is a perforation in the tympanic membrane [21].

Due to potential damage to the surrounding tissue, button batteries should be removed as soon as possible [13]. Spherical or sharp-edged objects are the most challenging to remove and require otomicroscopy-guided FB removal [13]. However, smooth and spherical FBs have the worst outcomes [13,22] because they cannot be readily grasped [22]. The removal of spherical objects is associated with the lowest success rate and the highest complication rate compared with the number of attempts [13].

According to Figueiredo et al., crucial factors contributing to complications are removal attempts by amateurs or untrained health professionals and inadequate hospital infrastructure. Moreover, they suggested the following steps to avoid possible complications: inform patients to seek an otorhinolaryngologist without delay in FB cases; increase awareness among otorhinolaryngologists of the technical challenges of removing FBs and improve education about urgencies in otorhinolaryngology graduate courses [6]. Furthermore, a study by Shih et al. indicates that pediatric aural FBs are more effectively and safely removed by otorhinolaryngologists compared to other medical professionals [19].

## 3. Nasal Foreign Bodies

**Figure 3:**
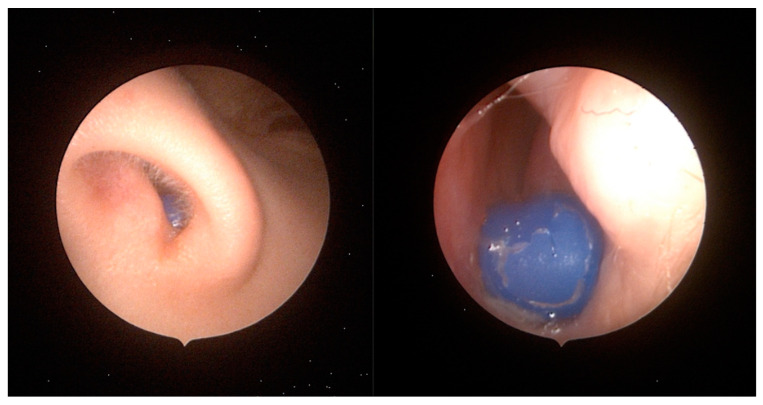
Endoscope view of a silicone pencil tip in the left nostril of a 10-year-old boy that was successfully removed with a hook.

Nasal FBs present another common emergency and account for approximately one-third of all otorhinolaryngological FBs [23]. Nasal FBs are almost exclusively found in children [6] and are more frequent than aural FBs [7]. The average age is around three years in most studies [11,18,23], with a male predominance [11,15,23]. This age distribution corresponds to their psychomotor development [23]. Most FBs are inorganic, accounting for 72–80% of extracted objects. Plastic beads or balls are the most common, followed by fragments of plastic or toys, foam pieces, rubber, pebbles, paper and cotton wool (Figure 3). Organic FBs are less frequent [24,25]. The most common location to lodge is on the right side, anterior to the middle turbinate or under the inferior one [16]. The right-side predominance may be due to a preference for right-handed children to put objects into their right nostrils [15,17,25]. This type of FB is diagnosed early because the incident is usually witnessed by adult caretakers [15], the child reports the presence of the FB or the parents discover it during washing or providing other care [23].

Most of the children are asymptomatic at admission, and those who are symptomatic usually have local pain, unilateral purulent nasal discharge associated with unpleasant nasal odor, epistaxis, the sensation of swelling or difficulty breathing [11,23]. Organic nasal FBs have swelling ability and are usually more symptomatic than inorganic [15]. Insects should be anesthetized and paralyzed with saline solution or xylocaine spray before extraction [26].

The minority of nasal FBs are diagnosed only when complications occur. The complication rate is about 12%, with epistaxis being the most common complication, followed by unilateral foul odor nasal discharge, nasal vestibulitis and mucosal irritation [15]. Lesions induced by nasal FBs vary according to the type of object and the lodge time [23]. The local inflammation of nasal mucosa may result in pressure necrosis followed by mucosal ulceration and erosion into blood vessels, producing epistaxis or septal perforation [15]. Secondary sinusitis is one of the possible complications caused by obstructed sinus drainage due to swelling [15]. Rhinolithiasis is a rare complication caused by the more prolonged presence of an FB in the nose [25,27,28].

The most concerning nasal FB is a button battery that can corrode and release caustic material, damaging the nasal tissue, causing necrosis and subsequent nasal septal perforation [15,18]. According to the literature, only 7 h is enough for septal perforation [29]. Magnets are another common inorganic object with a high rate of complications. Magnets in both nostrils or a magnet on one side and a ferrous object on the other can also cause septal perforation if not removed [18].

Most FBs can be removed without any anesthesia. However, general anesthesia is required in an operating room for some cases when the child is uncooperative, or complications have already occurred [15]. Removal is divided into positive pressure and mechanical extraction [30]. Positive pressure uses force to “blow” the object out while obstructing the opposite nostril. This is often impossible in younger children due to incompatible cooperation [18,30]. In that case, there is a common technique termed the “parent’s kiss” or “mother’s kiss” whereby the patient’s trained parent or trusted adult caregiver blows into the child’s mouth while occluding the unaffected nostril [31,32,33]. The air pressure during this technique is low, around 60 mm Hg; therefore, barotrauma is not a concern [31]. Other similar positive pressure techniques include using air from a mechanical device, either an Ambu bag or applying high flow (10–15 L/min) wall oxygen to the unaffected nostril using a tight-fitting tube or catheter [30,34]. Instilling a nasal decongestant before any attempts at removal is recommended [18,30]. There is always a risk of further displacement of the object into the nasal cavity and possible aspiration [30], so it is recommended to perform these techniques only for well-visualized and anteriorly placed objects [18].

Mechanical extraction includes several techniques and various tools. The proper preparation is essential, consisting of appropriate visualization and immobilization to prevent possible complications. A nasal decongestant is recommended only if there is no high risk of displacement further to the posterior. In some cases, topical analgesia is recommended before attempting extraction. The usual tools for mechanical removal include a nasal speculum, FB hook, straight or alligator forceps, balloon catheters and magnets. The use of these tools depends on the type, shape and location of the nasal FBs [18].

## 4. Pharyngeal Foreign Bodies

Almost half of pharyngeal FBs are present in children between 4 and 8 years of age [11]. No significant difference exists in the distribution of male and female children with pharyngeal FB. The most common symptoms of pharyngeal FB are local pain, gagging, dysphagia, vomiting and drooling. Common FBs include food, balloons, plastic bags and toys [21]. The most common pharyngeal FB is a fish bone, especially in regions surrounded by rivers and coastlines [11,35]. In children, fish bones are usually stuck in one of the palatal tonsils due to a narrow pharynx and large tonsils [36,37,38]. Typical examinations used to diagnose this condition include: oropharyngeal, radiological or endoscopic [35]. Extraction is performed under the obligatory proper visualization with different kinds of forceps.

In some cases, FB can cause the complete obstruction of the upper airway. Patients may then present with dyspnea, stridor or respiratory failure. In this case, immediate airway techniques such as the Heimlich maneuver, jaw thrust and clearing of the nasal or oral airways must be initiated. If these maneuvers do not improve the respiratory status, the following procedures may be used: directoscopy, with extraction of the FB using Magill forceps; endotracheal intubation, if possible; and urgent tracheotomy, in cases where all else fails [21].

## 5. Laryngeal Foreign Bodies

The symptoms of laryngeal FBs are determined by the FB’s size, shape, nature and obstruction degree [39,40,41]. Complete obstruction of the larynx causes asphyxia and, eventually, sudden death [42]. Round, ovoid and flexible objects are more likely to occlude a child’s airway [39,41]. Partial obstruction is much more common [21]. It is caused by smaller objects, which cause variable airway symptoms, including hoarseness, dysarthria, aphonia, stridor, cough, dyspnea, cyanosis and hemoptysis [21,39,43]. Clinical history is crucial to establish the diagnosis early because aspiration history is positive in more than 90% of cases [44,45]. The most common symptoms are a cough and hoarseness, which may mimic other respiratory diseases. Biphasic stridor or aphonia, as symptoms, decrease the risk of misdiagnosis and could present with the characteristic signs of aspiration [45].

If unusual symptoms of upper respiratory distress occur, a radiological review of all areas of the airways should be obtained. However, negative radiographic findings could not permanently eliminate the possibility of radiolucent FB [45]. Only a minority of children with laryngotracheal FBs had abnormal chest radiograph findings, and none of the laryngeal FBs were identified on chest X-rays and fluoroscopy [39]. Chen et al. considered that the involvement of a large proportion of radiolucent materials is the predominant reason for radiographic abnormality [45]. CT scans are increasingly used to image radiolucent FBs as CT is a more sensitive modality than conventional radiography [46].

Flexible laryngoscopy is an essential method by which FB can be clearly visualized and rapidly diagnosed. High suspicion indicates direct laryngoscopy or rigid bronchoscopy, the gold standard in diagnostics and treating aspirated FBs [45].

In the case of misdiagnosis, or if the FB remains within the respiratory tract for any reason, the complication risks increase with time [45]. According to Chen et al., nonspecific symptoms, a lack of FB aspiration history, delayed doctor visits and negative roentgenologic findings are independent risk factors for the misdiagnosis of laryngeal FB [45]. Patients with FBs admitted 24 h after the onset of the symptoms have a lower removal success rate than those who present earlier [11]. The most frequent complications are the formation of laryngeal granulation tissue and subglottic stenosis [45].

## 6. Tracheobronchial Foreign Bodies

**Figure 4:**
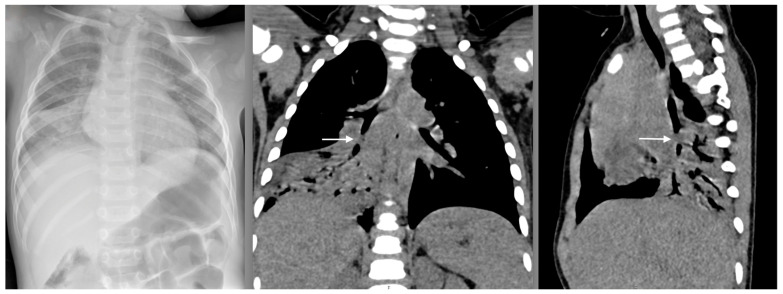
The initial X-ray finding of partial atelectasis of the middle right lung lobe in a 22-month-old boy (**left**). A CT scan revealed complete obstruction of the intermediate bronchus with a foreign body (peanut) (**middle** and **right**) that was successfully removed by rigid bronchoscopy.

FB aspiration is expected in early childhood, especially between 1 and 4 years of age, when it is also the leading cause of accidental death [47,48,49,50,51,52]. The peak incidence occurs between 12 and 24 months of age [53,54,55]. This is because of the young age, the hand-to-mouth technique of exploring the environment, ineffective chewing and possible swallowing disorders [56]. Again, there is a male predominance [49,57]. Most FB aspirations are witnessed by a caregiver [58]. The pediatric population is far more vulnerable to FB aspiration due to the narrow airway diameter, which can easily be obstructed. Most FBs are either partially or entirely ejected by coughing and spitting reflexes. Commonly aspirated FBs by younger children are organic materials such as nuts, seeds, vegetable matter or dried fruits. In contrast, inorganic materials such as toy pieces and pins are mostly aspirated by older kids [59,60]. Nuts and peanuts are the most aspirated objects [57,61]. Inflatable toys/objects are the most lethal airway FBs. Round objects are most likely to cause complete airway obstruction and asphyxiation. Other factors that make FBs more hazardous include compressibility and smooth and slippery surfaces [56]. FBs are commonly sufficiently small to pass to the trachea, but some lodge within it due to their wide caliber [62]. Most aspirated objects lodge in the bronchial tree, with the right main bronchus being the most common location because of its straighter trajectory relative to the trachea (Figure 4) [48,50,61,63,64]. Na’ara et al. found a right-side predominance only for infants, while Ding et al. found a predominance of the left side for children younger than two years and the right side for older children [58,65]. The mortality rate for pediatric airway FBs is 2.4%, significantly higher among children with chronic neurologic, cardiac and pulmonary disorders than in the healthy [66].

The signs and symptoms of airway FB vary according to the location of the lodgment [67], and that location varies according to the patient’s age and affects the complication rate and mortality [66]. An airway FB typically presents with at least one of the following symptoms at the time of aspiration: coughing, choking, stridor and/or wheezing [68,69,70]. This history and the occurrence of asymmetric breath sounds suggest an airway FB, but only 57% of cases have the classic clinical triad of sudden onset (choking/coughing, wheezing and unilaterally decreased breath sounds). These signs and symptoms have been shown to offer little predictive value [51,71,72]. Therefore, the FB may not be suspected without a history of these symptoms and may stay on-site longer [62]. Furthermore, the signs and symptoms of FB aspiration simulate several pediatric diseases, such as bronchiolitis, pneumonia and asthma, which can also lead to delayed diagnosis [65]. Other reasons for delayed diagnosis might include presenting subtle or nonspecific symptoms of lower airway FBs [69,70] and complications from non-indicated treatments such as steroids, antibiotics or bronchodilators [73,74]. Additionally, transfer of care from another hospital and hospital admission on the weekend affect the time before airway FB removal and its outcomes [66]. A long-retained tracheobronchial FB may cause the production of arachidonic acid, which results in an abnormal secretion of mucus and exudate that clogs up the lungs [75]. Furthermore, it can lead to chronic pulmonary infection, bronchiectasis, asthma, lung collapse or abscess [50,76,77]. Thus, patients may also present with fever and other signs and symptoms of pneumonia [62,78]. Pneumomediastinum is rare but one of the most severe and life-threatening complications of a retained tracheobronchial FB [61].

Two-view chest and lateral neck radiographs remain the initial diagnostic tests of choice [79], even though they depend upon the FB’s radiopacity and the degree of airway obstruction [49]. The sensitivity of chest radiography in diagnosis varies among studies from 61% to 88%, and the specificity from 30% to 97% [49,80,81,82]. The minority of aspirated objects are radiopaque; hence, the FB is visible on a chest radiograph in only 10–20% of cases [49,50,83]. Radiolucent objects are detected with standard radiographs only when aspiration is accompanied by airway obstruction or other complications, making diagnosing more difficult [53,84]. The most common signs on chest radiographs with an FB present are obstructive emphysema and atelectasis [49,67,84]. Rarely, pneumothorax or pneumomediastinum due to bronchial perforation or alveolar rupture may also be present [85]. Thus, chest radiography, with its high predictive value, can independently predict FB aspiration, but it cannot rule out an airway FB in 30% of cases with normal findings [53,70,86,87,88]. CT is a diagnostic option for asymptomatic or symptomatic but stable patients with normal or inconclusive plain radiographs and continuous clinical suspicion of FB aspiration (Figure 4) [89,90]. According to a recent systematic review and meta-analysis by El Khoury et al., chest CT has a sensitivity of 99% and a specificity of 92% for the detection of airway FB [91]. However, many cases can be definitively diagnosed only by performing a bronchoscopy [66]. Thus, high clinical suspicion must be maintained in patients with an aspiration history [72].

**Figure 5:**
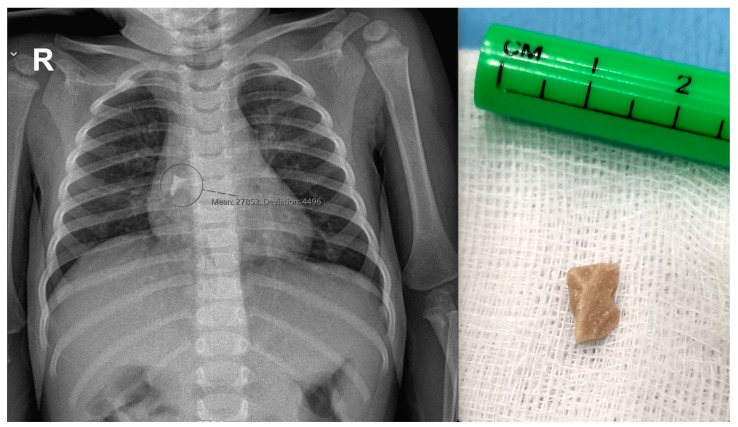
X-ray finding of a stone in the middle lobar bronchus, just below the branching of the right main bronchus of a 15-month-old girl (**left**), and the stone after successful removal with forceps using rigid bronchoscopy (**right**).

Rigid bronchoscopy is a method of choice to identify and extract the object in symptomatic cases [85,92,93]. It is indicated in symptomatic FB aspiration within 24 h of the event [94,95]. It allows airway control, good visualization, manipulation of the object with different forceps and rapid management of mucosal hemorrhage (Figure 5) [60,96]. This technique removes approximately 95% of FBs, and the currently reported complication rate remains below 1% [53,57,63,87,97]. In case of suspicion of multiple diminutive FBs or fragments, it is recommended to perform a flexible bronchoscopy after extraction to evaluate the entire tracheobronchial tree [98]. The most common postoperative complications after rigid bronchoscopy are hypoxemia, tracheal or bronchial laceration or bleeding, laryngeal edema, broncho-laryngospasm, pneumothorax, pneumomediastinum, reintubation, mechanical ventilation, pneumonia, tracheostomy, cardiac arrest and anoxic brain injury [99]. In a study by Zhang et al., complications occurred in 9% of cases, and hypoxemia was the most common postoperative complication [57]. Fragmentation or dislodgement of all or a part of the FB into the contralateral main bronchus is a potentially lethal complication if the previously involved bronchus remains obstructed by residual FBs or inflammation [100]. Fidkowski et al. reported the rate of major bronchoscopy complications of 0.96%, with a mortality rate of 0.42% in a study of 12,979 analyzed cases of pediatric airway FBs [48]. Postoperative adverse events and prolonged hospitalization are associated with preoperative cardiopulmonary compromise, ASA class 3 or 4, and prolonged operative time [99].

Flexible bronchoscopy can also be a safe diagnostic and treatment procedure with minimal risks and complications [67,101,102,103,104]. It has been widely used as the method of choice to remove pediatric tracheobronchial FBs. In such cases, FBs can be collected using biopsy forceps, snares and baskets [67,101,102]. In the case of non-sharp FB aspiration, with enough space between the object and the airway wall, the balloon method via flexible bronchoscope has proven to be a safe, effective and easily performed procedure for removal [105]. The main disadvantage of flexible bronchoscopy for FB extraction is the danger of dislodging and further compromising the airway [96].

Delayed diagnosis causes more common severe post-bronchoscopy complications that create significant morbidity [106,107]. Therefore, an awareness of the importance of early diagnosis in all health professionals is essential to reduce mortality and morbidity [45]. Furthermore, a standardized age-appropriate equipment list and staff training in the use of this equipment by otorhinolaryngologists is required to minimize patient morbidity and mortality [108]. On the other hand, passive intervention approaches to reduce the risk of FB aspiration, such as regulation that eliminates choking hazards from the market and persistent parental education can prevent child exposure and decrease morbidity [45].

## 7. Esophageal Foreign Bodies

Pediatric FB ingestion is a common and serious problem worldwide, particularly among children between 6 months and three years old [109]. The consequence is most likely due to infants exploring their surroundings primarily by hand-to-mouth activity [110]. Therefore, they commonly swallow small, shiny items like coins, pins and toy parts [111]. Most of these cases are witnessed by a caregiver and then, primarily within 48 h of ingestion, brought to medical attention [47,112,113,114,115,116].

The proximal esophagus is the narrowest portion of the pediatric alimentary tract and is the most common site for lodged FBs [117]. Most retained objects are coins [114,117,118,119], which show a slight male predominance [112,113,114,116,117,118,119]. There is also an increased risk of esophageal FB impaction in children with neurodevelopmental delay or underlying structural or motility abnormalities of the esophagus, such as stenosis [114,118,120,121].

Symptoms vary with the patient’s age, location and size of the FB. Infants may show nonspecific symptoms such as drooling, gagging or poor feeding. Older children may report odynophagia, dysphagia and chest pain [122]. Symptoms such as FB sensation sometimes result from a spontaneous FB pass, leaving superficial mucosal damage, e.g., fish bones [116].

In severe cases, FB ingestion can be life-threatening; prompt diagnosis and endoscopic removal are crucial to prevent morbidity and mortality. However, the diagnosis may be difficult due to delayed presentation or misdiagnosis due to nonspecific or lack of symptoms [116]. The physical examination includes airway and oropharyngeal evaluations, neck and upper thorax palpation to assess for crepitus and auscultation of the lungs [122]. An essential part of the diagnostic is a chest X-ray. Most esophageal FBs are radiopaque, but a normal chest radiograph cannot rule out an FB [117]. CT has a specificity of 96% and a high negative predictive value when the endoscopy is negative, so it is recommended if the patient is still symptomatic after a negative endoscopy [123].

Most FBs pass through the gastrointestinal tract without incident. Still, those retained can cause obstruction, tracheal compression, erosion, superficial abrasion, bleeding, esophageal stenosis, perforation, retropharyngeal abscess, tracheo-esophageal fistula and aorto-esophageal fistula [37,73,112,113]. Notably, impaction, perforations and obstructions usually occur at one of three anatomical constrictions of the esophagus [110], and FBs are primarily lodged in the cricopharyngeal sphincter [116,117,118,119]. However, complications are uncommon, caused mainly by retained food, coins and batteries, and are more likely to occur when the object has been impacted for a prolonged period [113,124]. The complication rate is directly proportional to the lodge time of the FB [125] and is the main reason why FB should not remain in the esophagus for more than 24 h after presentation [126].

It is thought that 80–90% of FB ingestions will pass without intervention, 10–20% will require endoscopic removal, and 1% will require surgical intervention [127,128]. Witnessed FB ingestion, visible objects on imaging and/or a high clinical suspicion indicate esophagoscopy [116]. Both rigid and flexible endoscopy are effective in FB removal, but the reported complication rate is higher for rigid devices. However, rigid esophagoscopy under general anesthesia is preferable for children and can be an alternative to flexible esophagoscopy [129]. Other reported modalities for FB removal include esophageal bougienage and balloon extraction under fluoroscopic guidance [117]. The most common postoperative complications are laryngeal edema, mucosal laceration and aspiration [130].

**Figure 6:**
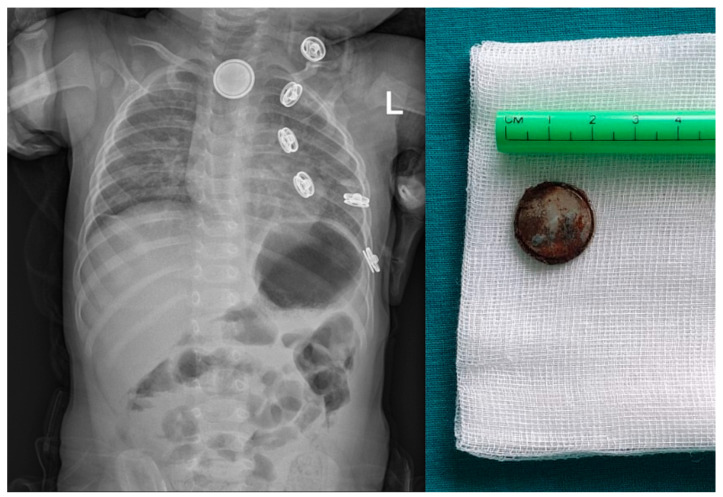
X-ray finding of a button battery in the proximal esophagus of an 11-month-old boy (**left**) and the corroded negative pole of the battery after successful removal with forceps using rigid esophagoscopy (**right**).

Over the last decade, children have increasingly swallowed more hazardous objects, such as button batteries, due to their widespread use as power supplies in electronic devices [111,131]. According to Buttazzoni et al., up to 61% of children who swallow button batteries develop serious complications [132]. It causes severe tissue damage due to sodium hydroxide buildup because of the electrical current discharged from the battery [131]. Damage can occur as early as 2 h after ingestion [128]. Furthermore, by physical pressure, lodged batteries, especially those with a diameter of 20 mm or more, can also cause tissue trauma and necrosis [133,134]. Even a discharged battery can cause this effect [135]. Lithium batteries are the only kind that produces complications, while alkaline batteries have no complications [136]. Radiographically, they can be distinguished from coins due to visible double-density circular opacity (Figure 6) [137]. Recent literature and guidelines support using honey after button battery ingestion in children over one year before endoscopic removal. This is due to its ease of application, minimal risk and proven ability to reduce mucosal injury [138,139,140]. It is recommended to use it with sucralfate only within 12 h of ingestion while waiting for endoscopic removal [139]. However, further studies are needed to clarify the effectiveness of honey treatment and the optimal treatment intervals completely [138]. In addition to using honey, it is advised to rinse with acetic acid after removing the button battery. The acid’s neutralizing action stops the further progression of mucosal necrosis [140,141,142]. Appropriate management is time-critical, and immediate endoscopic removal should be arranged within 2 h, generally not later than 6 h [135,143]. Otherwise, severe complications may occur, including aorto-esophageal fistula, vocal cord paralysis, perforation, hemorrhage and death [132,144,145,146]. In case of delayed diagnosis, more than 12 h after ingestion, it is recommended to perform a CT scan to evaluate possible vascular injuries before removal [139].

## 8. Conclusions

Otorhinolaryngological FBs and suspicions of them in the pediatric population require prompt and adequate diagnostics. The method of treatment depends on the location and type of foreign body. Furthermore, it should be performed by an experienced health professional or otolaryngologist without delay to prevent the development of possible complications. However, prevention is the best option; therefore, the greatest possible attention should be paid to toys and food within reach of children, bearing in mind their age, by parents, babysitters and teachers. The manufacturers of toys and food should necessarily highlight the potential danger for children on the product label. Therefore, there is a need for education about foreign bodies at all levels of society that include children in their scope.
